# Golden sea cucumber extract revives glucose transporter-4 and interleukin-6 protein level in diabetic mouse muscle

**DOI:** 10.14202/vetworld.2019.684-688

**Published:** 2019-05-18

**Authors:** Bambang Purwanto, Sundari Indah Wiyasihati, Putri Ayu Masyitha, Kristanti Wanito Wigati, Irfiansyah Irwadi

**Affiliations:** 1 Department of Physiology, Faculty of Medicine, Universitas Airlangga, Indonesia; 2 School of Medicine, Faculty of Medicine, Universitas Airlangga, Indonesia

**Keywords:** diabetes, glucose transporter, interleukin, muscle

## Abstract

**Background:**

Streptozotocin (STZ)-induced free radical oxidant activity resulted in muscle wasting due to protein carbonyl (PC), glucose transporter-4 (Glut-4), and interleukin-6 (IL-6) protein alteration. Antioxidant ingredient in the golden sea cucumber extract was found in promising level to inhibit free radical activity.

**Aim:**

This study was aimed to investigate the effect of golden sea cucumber extract on PC, IL-6, and Glut-4 level of STZ-induced diabetes mouse.

**Materials and Methods:**

This study was performed using mice, which were grouped into non-diabetes, diabetes, and diabetes-treated extract groups. The golden sea cucumber was extracted using 70% ethanol, which was administered by oral gavage twice a day for 5 consecutive days.

**Results:**

The extract reduced PC level and improved muscle Glut-4 and IL-6 protein level of diabetic mouse.

**Conclusion:**

The extract of golden sea cucumber revived muscle Glut-4 and IL-6 protein level in protection against muscle wasting.

## Introduction

Diabetes is a rapid growth worldwide non-communicable disease in the past three decades. In 2015, the diabetes prevalence was 8.5% of the world population which increased 4 times since 1980. Diabetes was found 6.9% of all Indonesian population, Indonesian diabetes prevalence was seventh rank worldwide. Diabetes produced some deadly risk problems, such as visual loss, heart attack, and renal failure. It also developed some disability problems, such as diabetic foot and muscle wasting [1,2].

Oxidative stress was associated with muscle wasting during hyperglycemia [3]. Free radicals oxidized some functional proteins, including muscle interleukin (IL)-6 and glucose transporter-4 (Glut-4). Oxidized proteins were carbonylated and then destructed by proteasome proteolysis enzymes [4]. In diabetes mouse muscle, protein carbonyl (PC) level was significantly higher [3], but IL-6 and Glut-4 level were significantly lower than normal [5]. Both proteins are needed to revive muscle under diabetes stress [6,7].

Antioxidant and growth factor stimulant were needed to improve muscle protein level and function. Both were found as active ingredients of golden sea cucumber (*Stichopus hermanii*) whole extract [8-10]. It was widely used in traditional food and medicine among Indonesians [8,11]. It was widely used in the treatment of liver disease (such as hepatitis and cirrhosis), dyslipidemia, and ulcers. A systematic review of toxicology studies did not find any adverse effect on golden sea cucumber extract as a daily oral supplement for 6 months [12].

This study was aimed to investigate the effect of golden sea cucumber extract on PC, IL-6, and Glut-4 level of diabetic mouse muscle.

## Materials and Methods

### Ethical approval

All procedures were approved ethically under supervision of the Committee of Health Research Ethic, Faculty of Medicine, Universitas Airlangga, letter number 228/EC/KEPK/FKUA/2018.

### Golden sea cucumber extract

Golden sea cucumber (*S. hermanii*) extract was obtained from the deep water of Makassar coastal, South Sulawesi, Indonesia. Fresh golden sea cucumber was then extracted at the Natural Science Centre of Laboratories, Brawijaya University, Indonesia. It was extracted using 70% ethanol and dried to achieve the optimum level of active substances. The process resulted in only 15 g extract from 800 g of golden sea cucumber. For each 100 mg of golden sea cucumber, extract contained 0.16 mg flavonoid.

The dose of golden sea cucumber extract was determined from the dose of flavonoid treatment at 10 mg/kg of body weight. Mouse with 20 g of body weight needed 0.2 mg of flavonoid or 125 mg of golden sea cucumber extract. The extract was dissolved into water and administered 2×1 ml/day by oral gavage to the diabetes-treated group mice, for 5 consecutive days. Water was used as a placebo that administered into non-diabetes (ND) mice and diabetes mellitus (DM) mice groups, 2×1 ml/day for 5 consecutive days.

### Diabetes animal model

Streptozotocin (STZ) was used to induce the diabetes-like model of mouse [13]. Previously, STZ injection impaired the muscle proteomic of the mouse in response to hyperglycemia. Muscle proteins were found wasted on STZ-injected mouse [5,6]. 27 male, 10 weeks old, 20±2 g of body weight Mus musculus BALB/c mice were grouped into ND, DM, and diabetes extract-treated (DM+extract) group. Serial STZ injection was used to induce hyperglycemia in diabetes and diabetes-treated group mice. Mice were injected i.p for 5 days successively using 40 mg/kg of body weight of STZ. The concentration of STZ was 22.5 mg/ml in citric buffer solution (pH 4.3). Blood glucose level was measured at day 10 of injection. The hyperglycemia was characterized by blood glucose level >300 mg/dl [3].

### Tissue processing for homogenate

Mouse calf muscle was obtained under anesthesia control, weighed, placed in the vacuum tube and soaked in 4°C poly buffer saline solution, then it was ground. The muscle homogenate was collected, transferred to Eppendorf, and centrifuged. The supernatant of homogenate was collected and measured for its PC, IL-6, and Glut-4 level. The concentration of each muscle homogenate was 33.33 mg/ml.

### PC measurement

PC level was measured using PC colorimetric assay kit (OxiSelect^™^ Cat no STA-315). The 2,4-dinitrophenylhydrazine solution was used as the reagent to measure PC level absorbance using 375 nm wavelength spectrophotometer. The PC level (nmol/ml) was determined using the standard curve of absorbance-PC level value. To obtain in nmol/mg muscle tissue, the level of PC was then divided by muscle homogenate concentration.

### IL-6 measurement

IL-6 level was measured using IL-6 enzyme-linked immunosorbent assay (ELISA) kit (Elabscience Cat E-EL-M0044). Ab anti-IL-6 was used to measure optical density value at 450 nm wavelength of ELISA reader. The optical density value was used to calculate IL-6 level (nmol/ml) in an equation of the standardized curve. The level of IL-6 was then divided by muscle homogenate concentration to get in nmol/mg muscle tissue.

### Glut-4 measurement

Glut-4 level was measured using Glut-4 ELISA assay kit (Elabscience Cat E-EL-M0564). Ab anti-Glut-4 was used to measure Glut-4 optical density value at 450 nm wavelength of ELISA reader. The optical density was used to calculate Glut-4 level (in nmol/ml) in an equation of the standardized curve. The level of Glut-4 was then divided by muscle homogenate concentration to get in nmol/mg muscle tissue.

### Statistical analysis

Data were collected and analyzed statistically for comparison between groups. Analysis of variance and its *post hoc* test were used to analyze the significance. The significance was determined with α<0.05.

## Results

### Characteristics data subject

Mice were characterized 10 days after STZ injection. There were significant differences between groups for body weight, muscle mass, and muscle glucose level. All those characteristics of mice were found lower in DM group compared to ND group. Characteristic of extract-treated group (DM+extract) of mice was not different from ND group. The comparison data between groups are shown in Table-1.

**Table-1:** The characteristic comparison between groups.

Groups	n	Body weight (g)	Muscle mass (mg)	Glucose level in muscle (mg/dl)
Non-diabetes	9	21.13±1.12^a^	354±12.25^a^	93.10±10.58^a^
Diabetes	9	18.41±0.96^b^	224±9.34^b^	52.79±21.15^b^
Diabetes extract treated	9	20.04±0.83^a^	318±14.37^c^	75.57±12.12^c^

Different superscripts (a, b, c) within the same column were statistically different at p<0.05. The characteristics were body weight, muscle mass, and glucose level of mouse after 5 consecutive days of treatment

Oxidative stress increased the level of carbonylated protein as the result of free radical oxidation in STZ-injected mice. PC level was found significantly higher at DM compared to diabetes-treated group and normal control (p=0.001).

Antioxidant activity of golden sea cucumber extract protected muscle proteins against free radical oxidant in diabetes. Glut-4 protein level was found lower in diabetic mice muscle compared to normal control (p=0.002). The treatment of golden sea cucumber extract improved Glut-4 level of protein significantly in diabetic mice muscle (p=0.017), but it was still lower compared to normal control (p=0.035). We also confirmed these findings with an improvement of glucose level and mass of muscle.

**Figure-1:**
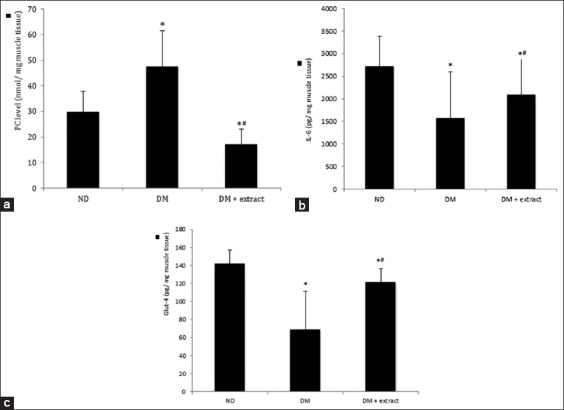
(a-c) protein carbonyl, interleukin-6, and Glut-4 level in mg gastrocnemius muscle tissue, comparison between groups. ND=Non-diabetes group, DM=Diabetes group, DM+extract=Diabetes extract-treated group. *Mean was significantly different to ND, ^#^Mean was significantly different to DM. Error bar indicates standard error for each group.

Golden cucumber extract also revived auto-paracrine inflammation control of IL-6. We found a significant improvement of muscle IL-6 protein level in diabetes-treated group compared to diabetes group (p=0.008). It was confirmed by a significant improvement of muscle mass. The comparison data of PC, Glut-4, and IL-6 level between groups are shown in Table-2 and Figure-1.

**Table-2:** Level of PC, IL-6, and Glut-4 in mg calf muscle tissue.

Groups	n	PC (nmol)	IL-6 (pg)	Glut-4 (pg)
Non-diabetes	9	29.91±8.36^a^	2719.44±1024.49^a^	141.91±15.64^a^
Diabetes	9	47.53±14.08^b^	1570.56±666.64^b^	68.57±43.76^b^
Diabetes extract treated	9	17.14±6.08^c^	2080.00±806.82^c^	121.47±15.67^c^

Different superscripts (a, b, c) within the same column were statistically different at p<0.05. PC=Protein carbonyl, IL.6=Interleukin 6 protein level, Glut.4=Glucose transporter 4 protein level

## Discussion

STZ is widely used to induce hyperglycemia diabetes-like model in rodents. STZ-induced beta pancreatic Langerhans cell damage thus impaired insulin stimulation for muscle metabolism [13]. Plasma insulin level reduced significantly after 5 consecutive days in mouse [14].

Insulin stimulates some intracellular signals for glucose uptake, glucose restoration, protein synthesis, and mass development in muscle. Impairment of insulin protein level induced muscle wasting in STZ diabetes-like mouse model [15]. Our findings confirmed that muscle glucose level, muscle mass, and body weight of DM mouse were lower than ND mouse (Table-1).

Insulin is needed to restore glucose for muscle mass development that influences body weight. Unfortunately, insulin treatment on STZ diabetes-like mouse was failed to improve body weight and muscle mass, even blood glucose level was significantly reduced. It might be another pathway that involved in muscle wasting on STZ diabetes-like mouse [3].

Vilela *et al*. [3] found a rise of free radical oxidant activities that induced oxidative stress. The marker of oxidative stress, malondialdehyde (MDA) level, was significantly higher after STZ injection. The activity of some intracellular antioxidant defense, such as reduced glutathione, glutathione peroxidase, and glutathione reductase, was also found significantly higher. Oxidative stress was associated with destruction in many tissues, especially in muscle. As oxidative stress marker, PC level, but not MDA, was associated with muscle wasting [4].

Our findings supported Vilela *et al*.’s [3] study (2016) that STZ injection increased oxidative stress marker of PC level. PC level was found significantly higher at DM group compared to DM+extract and ND group. Carbonylated protein altered the protein characteristics and its functions in the cytoplasmic milieu. PC lost its amine residue and is replaced by carbon residue, resulting in low water solubility. PC then precipitated and interrupted intracellular signaling. In massive destruction of muscle, PC was labeled and guided systemically to proteasome. Muscle lost its mass and function on metabolism and inflammatory control [4]. Antioxidant treatment on STZ diabetes-like model maintained mouse body weight and muscle glucose level [16]. Antioxidant treatment also reduced inflammation in STZ diabetes-like model [17]. Flavonoid, a natural antioxidant, was commonly used to treat STZ diabetes-like mouse model. Flavonoid was an active ingredient found in golden sea cucumber extract [11].

The antioxidant activity of flavonoid in golden sea cucumber extract was measured in the range of 50-55 μg/ml of half inhibitory concentration (IC_50_). Its antioxidant activity was categorized at a high level (IC_50_<100 μg/ml) [8]. Flavonoid was able to protect insulin receptor and Glut-4 protein against free radical oxidation [18]. Antioxidant activity of golden sea cucumber extract protected muscle proteins against free radical in diabetes [5].

Our findings confirmed the effect of golden sea cucumber extract which significantly lowered muscle PC level of DM+extract mouse compared to DM mouse. It also revived muscle function on glucose uptake and glucose restoration. The treatment of golden sea cucumber extract significantly improved Glut-4 level of protein in diabetes muscle, though it was still lower compared to the ND mouse. We also confirmed these findings with an improvement of muscle glucose level and muscle mass in DM+extract mice group.

The treatment of golden sea cucumber extract also improved muscle ability to control surrounding inflammation. Muscle secreted IL-6 in response to pro-inflammatory stimulation of macrophage tumor necrosis factor (TNF-α) [19]. Muscle IL-6 inhibited TNF-α signal transduction on inflammation, muscle wasting, protein degradation, and apoptosis [19,20]. We found a significant improvement of muscle IL-6 protein level in DM+extract mice group compared to DM.

Muscle IL-6 level was needed for myogenesis, myoblast proliferation, and differentiation. IL-6 was secreted higher as muscle responded to acute inflammation, but it was impaired as responded to chronic inflammation, such as in diabetes mellitus. Impairment of muscle IL-6 secretion induces proteolysis activity of proteasome, which leads to muscle wasting [20]. It was associated with a rise of free radical oxidant activity on diabetes muscle. The higher level of muscle IL-6 was found at lower muscle PC level of diabetes-treated golden sea cucumber extract.

## Conclusion

Golden sea cucumber extract revives muscle function on controlling glucose metabolism and inflammation due to its effect on the improvement of PC, IL-6, and Glut-4 protein level. Further study is needed to explore more about active substances beside flavonoid in the extract of golden sea cucumber which promises to be developed as new drug therapy for other diseases.

## Authors’ Contribution

PAM performed the experiment under the supervision of BP and II. BP designed the concept, analyzed, and interpreted the data. SIW, KWW, and II carried out the proofreading, guided the preparation, and finalized the manuscript for publication. All authors read and approved the final manuscript.
